# Genome-Wide Identification and Functional Classification of Tomato (*Solanum lycopersicum*) Aldehyde Dehydrogenase (ALDH) Gene Superfamily

**DOI:** 10.1371/journal.pone.0164798

**Published:** 2016-10-18

**Authors:** Jose C. Jimenez-Lopez, Francisco J. Lopez-Valverde, Paula Robles-Bolivar, Elena Lima-Cabello, Emma W. Gachomo, Simeon O. Kotchoni

**Affiliations:** 1 Plant Reproductive Biology Laboratory, Department of Biochemistry, Cell and Molecular Biology of Plants, Estación Experimental del Zaidín, Spanish National Research Council (CSIC), 1 Profesor Albareda, Granada, E-18008, Spain; 2 The UWA Institute of Agriculture, The University of Western Australia, 35 Stirling Highway, Crawley, Perth, WA, 6009, Australia; 3 Department of Biology, Rutgers University, 315 Penn St, Camden, New Jersey, 08102, United States of America; 4 Center for Computational and Integrative Biology (CCIB), Rutgers University, 315 Penn St, Camden, New Jersey, 08102, United States of America; Universidade Federal de Vicosa, BRAZIL

## Abstract

Aldehyde dehydrogenases (ALDHs) is a protein superfamily that catalyzes the oxidation of aldehyde molecules into their corresponding non-toxic carboxylic acids, and responding to different environmental stresses, offering promising genetic approaches for improving plant adaptation. The aim of the current study is the functional analysis for systematic identification of *S*. *lycopersicum* ALDH gene superfamily. We performed genome-based ALDH genes identification and functional classification, phylogenetic relationship, structure and catalytic domains analysis, and microarray based gene expression. Twenty nine unique tomato ALDH sequences encoding 11 ALDH families were identified, including a unique member of the family 19 ALDH. Phylogenetic analysis revealed 13 groups, with a conserved relationship among ALDH families. Functional structure analysis of ALDH2 showed a catalytic mechanism involving *Cys-Glu* couple. However, the analysis of ALDH3 showed no functional gene duplication or potential neo-functionalities. Gene expression analysis reveals that particular ALDH genes might respond to wounding stress increasing the expression as ALDH2B7. Overall, this study reveals the complexity of *S*. *lycopersicum* ALDH gene superfamily and offers new insights into the structure-functional features and evolution of ALDH gene families in vascular plants. The functional characterization of ALDHs is valuable and promoting molecular breeding in tomato for the improvement of stress tolerance and signaling.

## Introduction

Plants are frequently coping with different types of biotic and abiotic stresses during their life-cycle, i.e. dehydration, desiccation, cold and heat shock. This induces a rapid generation of reactive oxygen species (ROS), which consequently lead to accumulation of imbalanced cellular aldehyde levels which interfere with steady-state metabolic reactions in cells [1]. To cope with these stresses, plants have to express a broad spectrum of stress-responsive genes, which might play crucial roles in stress tolerance and survival [2]. Among these genes are aldehyde dehydrogenase (ALDH), enzymes that contribute to aldehyde molecules homeostasis as ‘scavengers’ to eliminate toxic aldehydes [3, 4]. The ALDH superfamily is a group of NAD(P) + -dependent enzymes that catabolize a broad spectrum of endogenous and exogenous aliphatic and aromatic aldehydes, as well as intermediates molecules or by-products derived from major metabolic pathways, by irreversible oxidation to carboxylic acids [5]. Beside these above mentioned activities, ALDHs also display several others functions such as (i) mediating in the secondary metabolism, particularly in the amino acid and retinoic acid metabolism; (ii) protection from osmotic stress through the generation of osmoprotectants molecules, i.e glycine betaine [5, 6]; and (iii) as other oxidereductase, ALDH enzymes produce NADPH and NADH contributing to redox homeostasis [7].

Most of the studied plant ALDH genes are highly expressed in response to salinity conditions, dehydration, heat, water logging, oxidative stress or heavy metals [8], suggesting crucial roles in environmental adaptation. In plants, the ALDH genes transcripts have been detected in various tissues and in response to different stressors [8, 9]. Thus, ALDH up-regulation is a common target of stress response pathway activation [10].

Sequencing genome projects are making available partial or entire genome sequences for the identification and comparative analyses of any gene family among species closely related or having extremely divergent adaptations. ALDHs are found throughout all taxa and have been classified into 24 distinct families based on protein sequence identities. ALDH superfamily has been identified in model plants as *Arabidopsis thaliana* [11], but also in other plant species [10]; these include the algae *C*. *reinhardtii* and *O*. *tauri*, the moss *P*. *patens* [12] and the vascular plants rice [13], maize [14], soybean [15], grape [16] and apple [17], but little is known about tomato ALDHs.

*Solanum* is a large angiosperm genus [18] that includes cultivated annual and wild perennial tomato plants from diverse environments. Tomato (*Solanum lycopersicum*) is one of the most important fruit crop for industry-related economy world-wide and a model system for fruit widely studied from different point of view, and the first crop to have a fully sequenced genome [19, Sol Genomics network: https://solgenomics.net/organism/Solanum_lycopersicum/genome].

Tomato genome sequencing project helped to identify and make the analysis of the ALDH gene families in the model plant for fruit. In this study, we systematically identified 29 ALDH genes belonging to eleven different families in the tomato genome, with the aim of studying their evolutionary relationship, expression profiles in different tissues and in response to various biotic and abiotic stresses by mining microarray datasets available to the public, as well as structure-functional features of the newly identified sequences of the ALDH family 2 and 3. The outcomes of the current study provided the groundwork for evolutionary and functional characterization of ALDH gene families in tomato and other plant species.

## Results and Discussion

### Characteristics of ALDH gene families in tomato

The completion and availability of the tomato genome sequencing, together with database searches allowed us to identify 29 ALDH gene sequences from tomato (Table 1), that code for members of 11 ALDH protein families (ALDH2, 3, 5 to 7, 10 to 12, 18, 19, and 22) which were previously identified in other plant species (Table 1), with the exception of ALDH19.

**Table 1:** Tomato ALDH genes and families.

Family	Gene Name	ALDH family member	Subcellular location	Molecular function	Signature	The Tomato Genome sequencing project / NCBI accession number	Reference
**Family 2**	SlALDH2B1	ALDH family 2 member B1	Mitochondrial	ALDH (NAD+)	E189-C223	Solyc02g086970.2.1	Current study
	SlALDH2B3	ALDH family 2 member B3	Mitochondrial	ALDH (NAD+)	E296-C330	Solyc05g005700.2.1	Current study
	SlALDH2B4	ALDH family 2 member B4	Mitochondrial	ALDH (NAD+)	E302-C336	Solyc08g068190.2.1	Current study
	SlALDH2B7a(variant 1)	ALDH family 2 member B7a	Mitochondrial	ALDH (NAD+)	E302-C336	XM_004236071.2	[57]
	SlALDH2B7b(variant 2)	ALDH family 2 member B7b	Mitochondrial	ALDH (NAD+)	E295-C329	XM_010317981.1	[57]
	SlALDH2B7c(variant 3)	ALDH family 2 member B7c	Mitochondrial	ALDH (NAD+)	E301-C335	XM_004245208.2	[57]
	SlALDH2B7d(variant 4)	ALDH family 2 member B7d	Mitochondrial	ALDH (NAD+)	E296-C330	XM_004238628.2	[57]
	SlALDH2C4	ALDH family 2 member C4	Cytosol	ALDH (NAD+)	E271-C305	XM_004251553.2	[57]
**Family 3**	SlALDH3F1a (variant 1)	ALDH family 3 member F1a	Cytosol	ALDH [NAD+/NAD(P)+];Variable substrate ALDH stress-regulated detoxification pathway activity;	-	XM_004228313.2	[57]
	SlALDH3F1b(Variant 2)	ALDH family 3 member F1b	Cytosol	ALDH [NAD+/NAD(P)+];Variable substrate ALDH stress-regulated detoxification pathway	-	XM_010319197.1	[57]
	SlALDH3F1c(Variant 3)	ALDH family 3 member F1c	Cytosol	ALDH [NAD+/NAD(P)+];Variable substrate ALDH stress-regulated detoxification pathway	-	XM_010319219.1	[57]
	SlALDH3F1d(Variant 4)	ALDH family 3 member F1d	Cytosol	ALDH [NAD+/NAD(P)+];Variable substrate ALDH stress-regulated detoxification pathway	-	XM_010318094.1	[57]
	SlALDH3H1	ALDH family 3 member H1	Cytosol, RE, Golgi, vacuole	ALDH [NAD+/NAD(P)+];Variable substrate ALDH stress-regulated detoxification pathway	-	XM_004241986.2	[57]
**Family 5**	SlALDH5F1a(variant 1)	Succinate-semialdehyde dehydrogenase	Mitochondrial	ALDH(NAD) activity;Succinate-semialdehyde dehydrogenase [NAD+/NAD(P)+]	E293-C327	NM_001246912	The Tomato Genome sequencing project
	SlALDH5F1b(variant 2)	Succinate-semialdehyde dehydrogenase	Mitochondrial	ALDH (NAD) activity;Succinate-semialdehyde dehydrogenase [NAD+/NAD(P)+]	E293-C327	NM_001306174.1	The Tomato Genome sequencing project
**Family 6**	SlALDH6B2	Methylmalonate-semialdehyde dehydrogenase [acylating]	Mitochondrial	ALDH (NAD+);(Methyl-) or Malonate-semialdehyde dehydrogenase	C320	XM_004230493.2	[57]
**Family 7**	SlALDH7B4a(variant 1)	ALDH family 7 member B4a	Cytosol	ALDH (NAD+)	E267	XM_010320171.1	[57]
	SlALDH7B4b(variant 2)	ALDH family 7 member B4b	Cytosol	ALDH (NAD+)	E267	XM_004235502.2	[57]
**Family 10**	SlALDH10A8	aminoaldehyde dehydrogenase	Peroxisomal	1—Pyrroline dehydrogenase activity;aminobutyraldehyde dehydrogenase activity	E260-C295	NM_001247306.2	[58]
	SlALDH10A9	aminoaldehyde dehydrogenase	Peroxisomal	1—Pyrroline dehydrogenase activity;aminobutyraldehyde dehydrogenase activity	E260-C295	XM_004241447.2	[58]
**Family 11**	SlALDH11A3a(variant 1)	NADP-dependent glyceraldehyde-3-phosphate dehydrogenase	Cytosol	Glyceraldehyde-3-phosphate dehydrogenase (NADP+) (non-phosphorylating)	E264-C298	XM_010324877.1	The Tomato Genome sequencing project
	SlALDH11A3b(variant 2)	NADP-dependent glyceraldehyde-3-phosphate dehydrogenase	Cytosol	Glyceraldehyde-3-phosphate dehydrogenase (NADP+) (non-phosphorylating)	E264-C298	XM_004242528.2	The Tomato Genome sequencing project
	SlALDH11A4a(variant 1)	NADP-dependent glyceraldehyde-3-phosphate dehydrogenase	Mitocondrial	Glycine decarboxylation via glycine cleavage system	-	XM_004244496.2	[57]
	SlALDH11A4b(variant 2)	NADP-dependent glyceraldehyde-3-phosphate dehydrogenase	Mitocondrial	Glycine decarboxylation via glycine cleavage system	-	XM_004244497.2	[57]
**Family 12**	SlALDH12A1	Delta-1-pyrroline-5-carboxylate dehydrogenase 12A1	Mitochondrial	ALDH (NAD+)	-	XM_004241521.2	The Tomato Genome sequencing project
**Family 18**	SlALDH18B1	δ-1-pyrroline-5-carboxylate synthase	Chloroplast, cytosol and membrane	δ-1-pyrroline-5-carboxylate synthetase activity;Glutamate 5-kinase;Glutamate-5-semialdehyde dehydrogenase	PS00902—PS01223	XM_004240639.2	The Tomato Genome sequencing project
	SlALDH18B2	δ-1-pyrroline-5-carboxylate synthase	Chloroplast, cytosol and membrane	δ-1-pyrroline-5-carboxylate synthetase;Glutamate 5-kinase activity;Glutamate-5-semialdehyde dehydrogenase	PS00902—PS01223	XM_010323651.1	The Tomato Genome sequencing project
**Family 19**	SlALDH19	γ-glutamyl phosphate reductase		γ-glutamyl phosphate reductase (Biosynthesis of proline)	PS01223		[27]
**Family 22**	SlALDH22A1	ALDH family 22 member A1	RE and extracellular region	ALDH (NAD+)	E294-C328	XM_004252875.2	The Tomato Genome sequencing project

The identification of tomato ALDH sequences has been done based on previously identified ALDH sequences from other species such as Arabidopsis-, rice-, maize-, grape-, soybean, *Sorghum bicolor*,- *Selaginella moellendorffii*-, poplar-,moss-, algae-, and *O*. *tauri*-, by retrieving these sequences and running BLAST searching as specified in Methods section. All putative ALDH sequences identified with E-value <1e-6 were manually analyzed to confirm the ALDH functional motifs Pfam00171 (ALDH 3 family), PS00687 (ALDH glutamic acid active site), and/or PS00070 (ALDH cysteine active site); in addition to the superfamily domains KOG2450, KOG2451, KOG2453, and KOG2456.

The classification of protein families were made according to AGNC [18], protein root symbols (ALDH) were followed by a family description number (2, 3, 5, 6, 7, etc.), a subfamily descriptor (A, B, C, D etc.) the individual gene number (chromosomal position within each subfamily), and a low-case letter to designate the number of variant (a, b, c, d, etc) as illustrated in Table 1.

The ALDHs identified in *S*. *lycopersicum* encode proteins ranged from 161 to 721 amino acids (aa) in length, with predicted isoelectric points (pIs) from 4.75 to 6.25. Seven families (2, 3, 5, 7, 10, 11 and 18) were characterized at least for one gene. We observed that families 5, 12, and 22, as well as members of the ALDH2C4 were defined by a single gene member in tomato, similar to Arabidopsis (S1 Table), probably because these three families constituted house-keeping ALDH genes, involved in central plant metabolism and preservation of nontoxic aldehyde levels. Three families (2, 3 and 11) were comparatively abundant in *S*. *lycopersicum*.

In comparison to Arabidopsis class 3 ALDHs, tomato lack ALDH3I1, but contained 5 more members, where some of them were suggested to be substitutes for the orthologous Arabidopsis ALDH3I1 function.

Family 2 was represented by 8 gene members in tomato and less in other plant species (with the exception of apple and cotton). Family 11 was represented by 4 gene members in tomato, 5 and 6 members in *P*. *patens* and *S*. *moellendorffii*, respectively, and lower numbers in the rest of species analyzed (S1 Table). Understanding biological implications of the presence of one ALDH member in some families (5, 12 and 22) and the various duplications in other ALDHs (2 and 11) is of substantial functional value. The main function of ALDH2 gene identified in plants is as nuclear restorer (rf2) of cytoplasmic male sterility (cms) [20]. On the other hand ALDH2 play an important role in detoxifying lipid peroxidation-derived aldehydes produced during oxidative stress [21] in mammals. However, ALDH2 specific functions of both mitochondrial and cytosolic proteins in plants remain to be determined. Thus, we can rule out that ALDH2 duplications may be implicated as fertility restorer, and/or implicated in different oxidative stresses as detoxifying molecules.

Therefore, ALDH11 family in Arabidopsis has a crucial function in the generation of NADPH for biosynthetic processes from photosynthetic glyceraldehyde-3-phosphate exported from the chloroplast [22]. Although duplication of genes from tomato ALDH11 suggests an increase in the NADPH synthesis that may be used for sugars production [23], other alternative functions remains to be investigated.

ScanProsite analysis showed that characteristic PS00687 and PS00070 domains were absent in some of the ALDH sequences. 15 out 29 genes contained PS00070 domains (ALDH cysteine active site) and PS00687 (ALDH glutamic acid active site), which are frequently found in the ALDH protein superfamily; five sequences contained only one of these domains (PS00687 domain was absent in ALDH19 and ALDH6; PS00070 domain was absent in ALDH3H and ALDH7). Although PS00687 or PS00070 domains were absent in some proteins, other searches for alternative conserved domains indicated that they belonged to the ALDH superfamily.

ALDH3F and ALDH12A did not contain these domains. However, after searching for functional domains within these two families of proteins in NCBI http://www.ncbi.nlm.nih.gov/ indicated that ALDH3F and ALDH12A still belonged to the ALDH superfamily. PS00902 domain (Glutamate 5 kinase signature) was present in two sequences (ALDH18), and PS01223 domain (γ-glutamyl phosphate reductase signature) was found in three sequences (ALDH18 and ALDH19).

All ALDH gene families identified in higher plants such as Arabidopsis were presented in *S*. *lycopersicum* (S1 Table). When compared to other well-characterized plant ALDH families, tomato is the third most abundant having 29 genes, compared to 39 in apple, 30 in cotton, 26 in Black cottonwood, 25 in grape, 23 in maize, 20 in rice, 16 in Arabidopsis (S1 Table). *S*. *lycopersicum* seem to have additional stress–response proteins among ALDHs, enabling it to tolerate environmental stress such as salinity, drought, i.e. gene numbers in particular ALDH families showed different variants as in ALDH2, ALDH3, ALDH5, ALDH7 and ALDH11. Thus, ALDH2, ALDH3 and ALDH11 are particularly large, which may be functionally important carrying out detoxification of aldehyde molecules generated under different stress, and maintaining the homeostasis of reducing equivalents. ALDH6, ALDH12 and ALDH22 were integrated only for one member in comparison to most of the species, including tomato.

Orthologous genes conceivably had identical functions, but tended to diverge in regulatory and coding regions which led them to alter the expression patterns and to acquire new functions, respectively [24]. In addition to the ALDH commonly shared molecular function (oxidation-reduction process), several member of the *S*. *lycopersicum* ALDH family members exhibit orthologous functional domains that have been also identified in other species. Table 2 summarizes other orthologous derived functions, where included cellular location, potential molecular functions, which are identified by an OrthoDB gene identification (ID) number. Overall, we have found seven alternative functional domains delivered among different members of the tomato ALDH families, which are implicated in DNA binding, i.e. ALDH11, and metabolic processes, i.e. ALDH3, or oxidative stress, i.e. ALDH6 and 12 (Table 2). Interestingly, searching in OrthoDB using tomato ALDH11A4 revealed an orthologous, 1-lipoyl-binding domain (ID: EOG09360), in multiple species, which is a glycine decarboxylation via glycine cleavage system located in mitochondria.

**Table 2:** Tomato ALDH orthologous genes. Identification number (EO) for potential orthologous functional domains is indicated in the third column with their respective names.

Family	Gene Name	Orthologous	Molecular Function	Biological Process	Cellular Component	Species
**Family 2**	SlALDH2B1	Armadillo-like helicalEOG0936062U/Ribosomal protein L37EOG0936062U	Proteins Binding/Oxidoreductase activity and structural constituent of ribosome	Mitotic chromosome condensation/Metabolic process	Plasmodesma/Intracellular	*Oryza meridionalis/Solanum tuberosum*
	SlALDH2B3					
	SlALDH2B4					
	SlALDH2B7a					
	SlALDH2B7b					
	SlALDH2B7c					
	SlALDH2B7d					
	SlALDH2C4	AAA+ ATPase domainEOG0936084C	Metalloendopeptidase activity/ATP binding activity	Proteolysis	Membrane	*Oryza meridionalis*
**Family 3**	SlALDH3F1c	Transmembrane: HelicalEOG09360822	oxidation-reduction process	metabolic process	-	*Oryza barthii/Oryza glumipatula/Oryza punctata*
	SlALDH3F1d					
**Family 6**	SlALDH6B2	Transmembrane: HelicalEOG09360822	Methylmalonate-semialdehyde dehydrogenase (acylating) activity/Copper ion binding	Response to oxidative stress/Metabolic process/Chlorophyll catabolic process	Integral component of membrane/Mitochondrion	*Aegilops tauschii*
**Family 11**	SlALDH11A3a	Homeodomain-likeEOG093607AG	DNA Binding	Transcription regulation/DNA-template	Nucleus	*Oryza meridionalis*
	SlALDH11A3b					
	SlALDH11A4a	1 lipoyl-binding domainEOG09360PSV	Glycine cleavage system H protein	Glycine decarboxylation via glycine cleavage system/Response to fructose, glucose, sucrose	Mitochondrial	*Aegilops tauschii/Amborella trichopoda/Arabidopsis lyrata/Arabidopsis thaliana/Brachypodium distachyon/Brassica rapa/Glycine max/Hordeum vulgare/Medicago truncatula/Musa accuminata/Oryza sativa/Populus trichocarpa/Prunus pérsica/Solanum lycopersicum/Triticum aestivum/Vitis vinifera/Zea may*
	SlALDH11A4b					
**Family 12**	SlALDH12A1	Transmembrane: HelicalEOG09360822	Oxidation-reduction process	Metabolic process	-	*Oryza meridionalis*
		P5CDH1EOG093605J6	Proline metabolic process	Reactive oxygen species metabolic process	Chloroplast	*Solanum tuberosum*
**Family 18**	SlALDH18B1	Transmembrane: HelicalEOG09360822	Glutamate 5-kinase activity/Copper ion binding	Proline biosynthetic process/Response to oxidative stress//metabolic process/chlorophyll catabolic process	Cytoplasm/Integral component of membrane//Mitochondrion	*Glycine max/Oryza sativa*
	SlALDH18B2					

### Phylogenetic analysis of tomato ALDH genes

**Fig 1:**
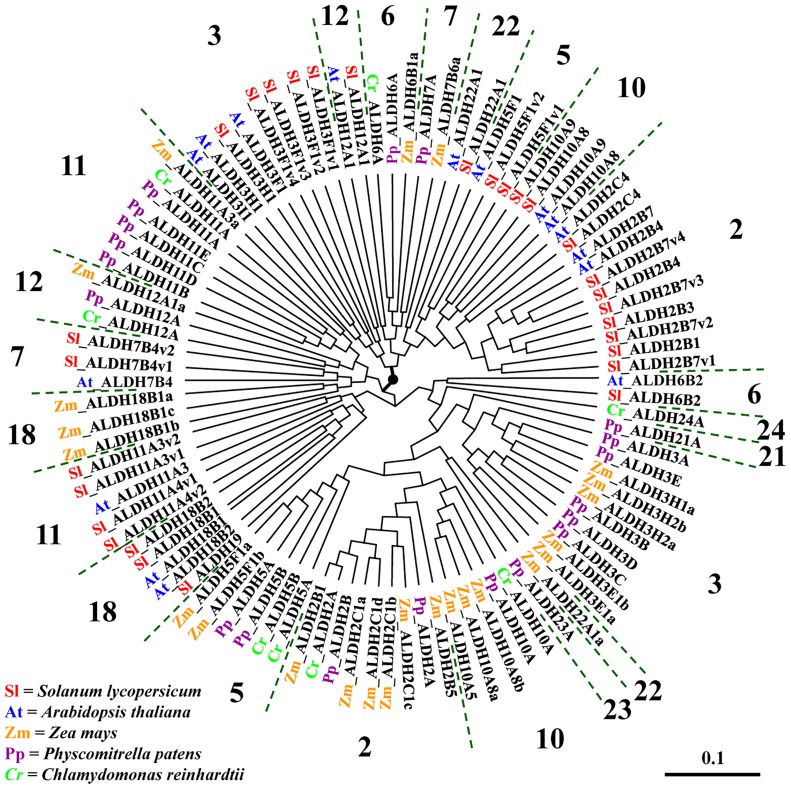
Comparative phylogenetic analysis of tomato ALDHs. Phylogenetic analysis was made using 97 ALDH proteins from *Solanum lycopersicum* (Sl), *Arabidopsis thaliana* (At), Zea mays (Zm), *Physcomitrella patens* (Pp), and *Chlamydomonas reinhardtii* (Cr).

In order to examine the phylogenetic relationship among tomato ALDH genes including the comparative analysis with the model plant Arabidopsis, 97 full-length ALDH protein sequences identified in *S*. *lycopersicum*, *A*. *thaliana*, *Zea may*, *Physcomitrella patens* and *Chlamydomonas reinhardtii* were aligned to further generate a phylogenetic tree. Fig 1 shows 23 groups with ALDHs from the same families from the different species clustered together. ALDH members of the same family did not always group together, which is the case of all families except for families 21, 22, 23, 24.

To understand the relevance of different functional members of ALDH protein families, we established phylogenetic relationships between ALDHs from *S*. *lycopersicum* and the well-studied *Arabidopsis thaliana*, but also with monocots (*Zea mays*), moss and algae. Phylogenetic tree (Fig 1) indicates that tomato ALDHs share a common plant ALDH family core mostly with Arabidopsis for all ALDH families. Among ALDH families, the most distantly related families in the phylogeny were ALDH19, ALDH11 and ALDH18 grouped in a well separate cluster. This finding is consistent with previous research in rice [13], maize [14], soybean [15], grape [16], Arabidopsis [7], indicating that these proteins had the greatest degree of sequence divergence from the other ALDH families and did not contain the conserved ALDH active sites [25]. A possible reason supporting this observation is that members of the ALDH11 family, a non-phosphorylating glyceraldehyde 3-phosphate dehydrogenase (GAPN; EC 1·2·1·9), catalyzes the irreversible NADP+-dependent oxidation of glyceraldehyde 3-phosphate to 3-phosphoglycerate and NADPH, which is the main source for mannitol biosynthesis in many plant species [23]. ALDH18 have high degree of sequence divergence from the other ALDH families and does not contain the exact generally conserved ALDH active sites [26]. A likely reason for this observation is that members of the ALDH18 family may be involved in a variety of biological processes, which require that a very diverse range of substrates can be recognized in a sequence- and/or structure-specific manner. ALDH19 only has been found in *S*. *lycopersicum* among higher plants, which has only been identified in the sequenced tomato genome and encoding a γ-glutamyl phosphate reductase. It catalyzes the NADP-dependent reduction of l-glutamate 5-phosphate to 1-glutamate 5-semialdehyde, which may perform a role in the biosynthesis of proline from glutamate [27]. Furthermore, family ALDH3 is also quite divergent since it integrates a group of isozymes that may play a major role in the detoxification of aldehydes generated by alcohol metabolism and lipid peroxidation. In Arabidopsis, ALDH3 might have evolved as a consequence of functional specialization in different tissues and subcellular compartments [28].

Higher plants like *S*. *lycopersicum*, *G*. *raimondii*, *M*. *domestica*, *V*. *vinifera* and *Z*. *mays*, seem to be more abundant in ALDH genes content in comparison to animals and fungi. Unlike mammals, plants are sessile and therefore more vulnerable to environmental stress factors. As a consequence, they may require additional mechanisms for stress-response like proteins such as ALDHs to protect them under abiotic and biotic stresses expositions [29]. In this regard, it was found that although glyophyte *Arabidopsis thaliana* and halophytes and halophyte *E*. *salsugineum* have equal number of ALDH superfamily members (Table 2), they have different expression patterns of ALDH7B4 and ALDH10A8 suggesting that *E*. *salsugineum* uses modified regulatory pathways, which may contribute to salinity tolerance [7].

Interestingly, the abundance of the ALDH genes in bryophytes such as *P*. *patens* may be linked to the transition from aquatic environment to amphibious life. This translated into an increased structural and developmental complexity, where additional genes were required to cope with environmental stresses during the environmental (aquatic to amphibious) transition [29]. On the other hand, plants completing their life cycles on land would lose several genes related to the aquatic life, as well as genes necessary for adaptation to land environmental life would be more abundant. These genetic events of gene loss and/or abundance would also occur in the ALDH superfamily.

Among the 24 ALDH families, plants ALDH are present in 14 families: ALDH2, and 3, ALDH5 to 7, ALDH10 to 12, ALDH18, and 19, ALDH21 to 24 (ALDH11, ALDH12, ALDH19, ALDH21 to 24) are unique to plants. So far, a single gene of the ALDH19 family has only been identified in tomato and it is also unique to plants [27], suggesting that ALDH19 may have evolved specifically in this lineage.

ALDH21 and ALDH23 have been only found in *P*. *patens* and *S*. *moellendorffii*, while ALDH24 is unique to *C*. *reinhardtii* (Table 2). The comparative study of *S*. *lycopersicum* to other vascular plants showed the ten common-shared core of ALDH families (ALDH2, ALDH3, ALDH5 to 7, ALDH10 to 12, ALDH18, and ALDH22), suggesting a previous evolution of these core of families to the monocot/eudicot divergence. Eight out of ten of these families (ALDH2, ALDH3, ALDH5, ALDH6, ALDH10 to 12, and ALDH22) are also commonly-shared by land plants and algae, suggesting an ancient origin for these families, even previously to the transition of aquatic plants onto land (Table 2). Remarkably, due to nomenclature mistakes after genes identification, ALDH1 and ALDH4 gene family members are not found in plants. In addition, ALDH1 and plant ALDH2 genes should be grouped together according to AGNC nomenclature guidelines. The same case applied to ALDH4 and ALDH12, which belong together to the single family since both encode δ-1-pyrroline-5-carboxylate dehydrogenases, which are involved in the degradation of proline to glutamate [30].

### Structure-based functional analysis of *S*. *lycopersicum* ALDH 2 and 3

**Fig 2:**
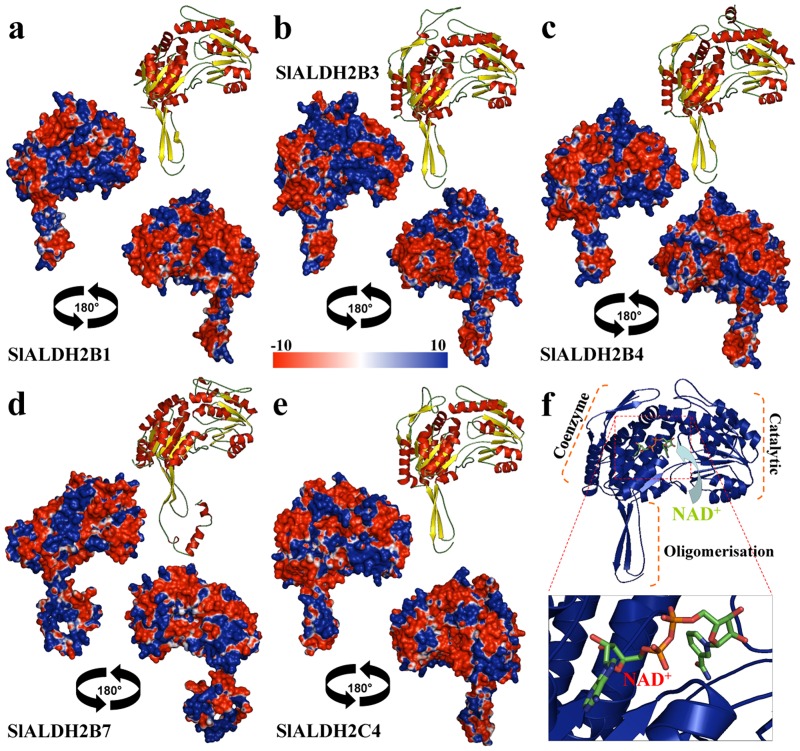
Structural analysis of *S*. *lycopersicum* ALDH2 superfamily. Three-dimensional structure of tomato SlALDH2 corresponding to the families (A) 2B1, (B) 2B3, (C) 2B4, (D) 2B7, and (E) 2C4. Structures were depicted as a cartoon diagram. α-helices, β-sheets and coils are depicted in red, yellow and green, respectively. Two views rotated 180 around the x-axis are provided for SlALDH2 superfamily of the electrostatic potential representation on the SlALDH2 protein surfaces. The surface colors are clamped at red (-10) or blue (+10). F) Detailed view of the SlALDH2 chain and the active (catalytic) site, and the spatial distribution of the coenzyme. Residues are depicted as stick and colored according with atoms.

**Fig 3:**
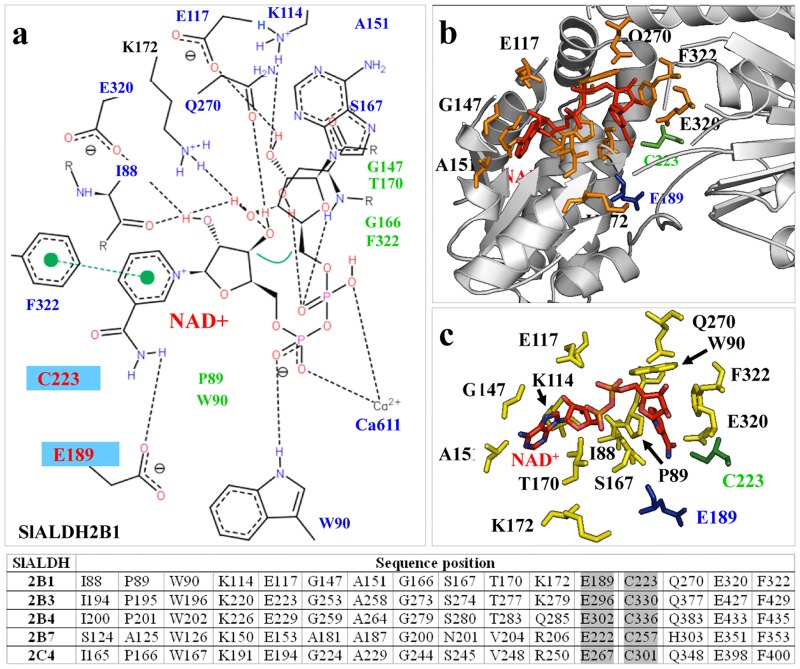
Coenzyme and ligand-binding domain analysis of ALDH2 superfamily. (A) Hydrogen-bonding interactions in the SlALDH2B1 coenzyme and ligand-binding domain and its interaction with NADH. Hydrogen bonds are shown in broken lines. Critical catalytic amino acids (C223 and E189) are highlighted in blue background. (B) Distribution of the NADH cofactor (red color) and the spatial distribution of the residues that integrate the cofactor-substrate binding cleft. Residues are depicted as stick and orange colored and green/blue colors the catalytic residues. (C) Detailed representation of the amino acids interacting with NADH and stabilizing the cofactor in the catalytic domain. Additional table shows the key amino acids involving the coenzyme-substrate binding domain. Catalytic crucial residues as grey color shadowed.

**Fig 4:**
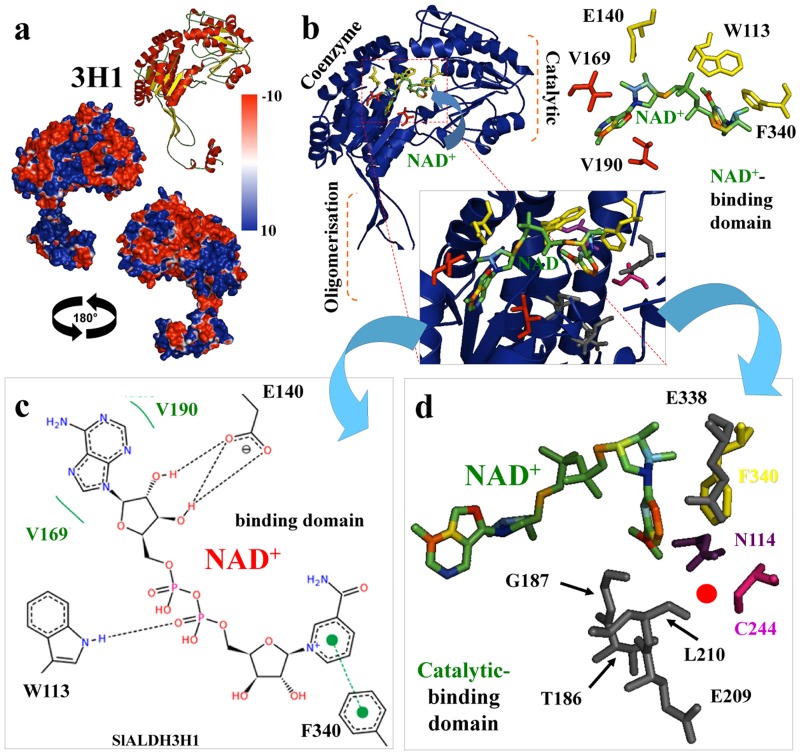
Tomato ALDH3H1 protein structure, coenzyme and ligand-binding domain analysis. (A) Three-dimensional structure of tomato SlALDH3H1. Structures were depicted as a cartoon diagram. α-helices, β-sheets and coils are depicted in red, yellow and green, respectively. Two views rotated 180 around the x-axis are provided for SlALDH3H1 superfamily of the electrostatic potential representation on the SlALDH3H1 protein surfaces. The surface colors are clamped at red (-10) or blue (+10). (B) Detailed view of the SlALDH3H1 chain and the active (catalytic) site, and the spatial distribution of the coenzyme the amino acids involved in holding NAD+. Residues are depicted as stick and colored according with atoms. (C) Hydrogen-bonding interactions in the SlALDH3H1 coenzyme domain and its interaction with NAD+. Hydrogen bonds are shown in broken lines. (D) Detailed view of the catalytic-binding domain showing residues configuring this domain, and critical catalytic amino acids (C244 and N114) highlighted in pink and purple color in proper position related to the substrate (red dot).

**Fig 5:**
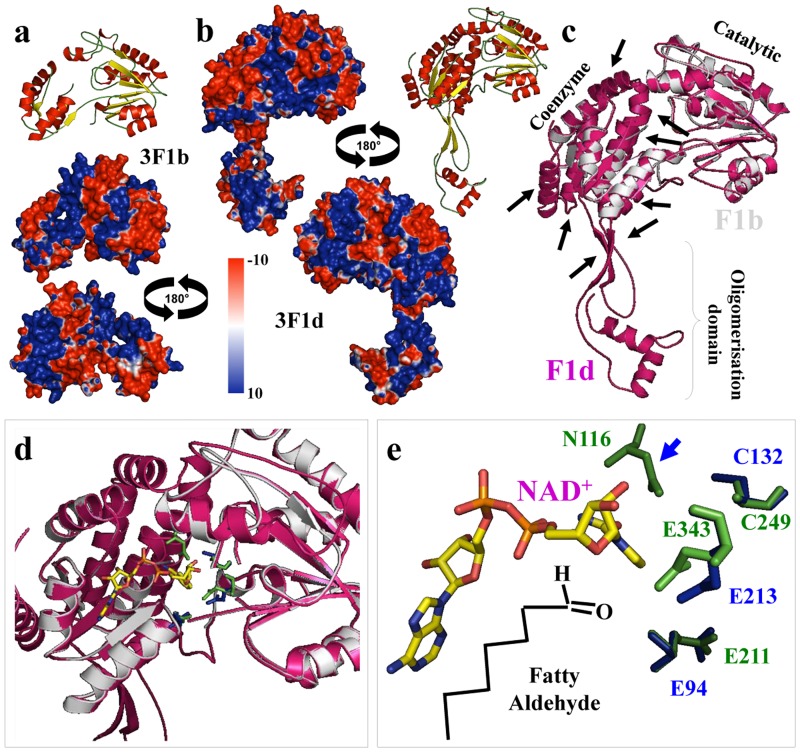
Tomato ALDH3F1 (b and d variants) proteins structure, coenzyme and ligand-binding domain analysis. Three-dimensional structure of tomato (A) SlALDH3F1b missing the complete oligomerization domain and partially the coenzyme domain; (B) SlALDH3F1d. Structures were depicted as a cartoon diagram. α-helices, β-sheets and coils are depicted in red, yellow and green, respectively. Two views rotated 180 around the x-axis are provided for both SlALDH3F1 protein variants of the electrostatic potential representation on the SlALDH3F1 proteins surfaces. The surface colors are clamped at red (-10) or blue (+10); (C) Superimposition of SlALDH3F1b and SlALDH3F1d showing the three main domains (coenzyme, oligomerization and catalytic), and highlighting with black arrows all the 2D-structural elements missing in SlALDH3F1b; (D) Detailed view of the superimposition showing the co-enzyme/catalytic cleft accommodating the NAD+ and substrate holding amino acids; (E) Detailed view of the catalytic-binding domain showing residues integrating this domain, and critical catalytic amino acids (C132 and N116) highlighted in green and blue colors in proper position related to the substrate (fatty aldehyde).

The ALDH gene superfamily has been explored in various organisms, mostly from a systematic point of view [7]. Solving the crystallographic structure of selected ALDHs and afterward being deposited in the Protein Database (PDB) has made possible to study the structure-functional related features of ALDH [13, 14]. To our knowledge, structure-functional homology modeling and the study of 2-D and 3D features comparative analysis of the complete ALDH protein superfamily have been only performed in few organisms such as rice [13] or maize [14]. Using computational homology modeling, we have uncovered the 3D structure features of the catalytic active sites and the NAD(P)+ -ring binding clefts of the *S*. *lycopersicum* ALDH2 (Figs 2 and 3), and ALDH3 (Figs 4 and 5) respective families. Each sequence entered in the protein structure modelling work-flow, where the best structural templates where used to build each domain of the proteins. When first model was obtained, it was refined (energy minimization and structural discordances) using the structural parameters summarized in material and methods.

The structural assessment of the accuracy of the ALDH2 and ALDH3 (H1 and F1) models were made throughout a comparative analysis to the templates (crystallographic structures PDBs accession numbers 4pxl, 4qgk, and 1ad3, respectively), which were used to build the models, and using stereo-chemical and energy minimization parameters displaying the following data:

The analysis of the best templates showed values of 0.83, 0.74, and 0.749 for the Q-mean parameter (linear combination of six terms, including stereology and energy, to estimate the model reliability ranging between 0 and 1), respectively, and 0.767, 0.752, and 0.674 for ALDH2 and ALDH3 (H1 and F1) models, respectively. Another parameter to check the overall quality of the structures, ProSA, showed a z-score of −9.19, -9.49, and -9.04 for ALDH2 and ALDH3 (H1 and F1) models, respectively; and −9.97, −10.31, and −9.58, respectively for the individual crystallographic structural templates. Both, Q-mean and ProSA parameters show values quite similar for the ALDH2 and ALDH3 (H1 and F1) models compared to the crystallographic structures, which mean that ALDH2 and ALDH3 (H1 and F1) protein models built are accurate and close to its templates in structure quality.

Therefore, we also analyzed the stereochemistry of the model using Procheck analysis (Ramachandran plot), showing that 92.1%, 88.7%, and 91.6% of the structural residues were located in favorable regions in the respective templates; 7.4, 11.0, and 7.9 in allowed regions, 0.2, 0, 0 in generally allowed regions; 0.2, 0.3, and 0.5% for the four templates in disallowed regions. These values for ALDH2 and ALDH3 (H1 and F1) models were 92.8, 6.1, 0.6, and 0.6%, respectively for ALDH2; and 92.3, 6.3, 0.5, and 1% / 90.4, 7.7, 0.9, and 0.9%, respectively for ALDH3 (H1 / F1), finding even more residues located in favorable regions, less residues in allowed regions, and a similar situation in generally allowed and non-favorable regions.

If we make an overall analysis by taking together all these parameters, in addition to the LDH2 and ALDH3 templates information, we confirm the accuracy and reliability of the structural models built for ALDH2 and ALDH3 (H1 and F1) proteins, in the basis of their crystallographic templates. Thus, these protein models can be perfectly used for further structure-functional analyses. A similar assessment was made for the other structures built for ALDHs with similar results when compared to their templates.

The major differences found in the structure of ALDHs were located in the oligomerization region, where parameters as length, number of 2D elements, curvature angle of a-helices and b-sheets, and folding characteristics were prominent. ALDH2B7 exhibited a long loop, and coenzyme domain of the ALDHs (Fig 2A–2E), particularly for ALDH2B7 [31], but the overall topology was quite similar among members of the same family. We also found that the catalytic domain was quite similar along the ALDH2 superfamily, as a detailed view is displayed in Fig 2F. However, curvature angles of the coils structures exhibited the largest differences when the catalytic domain was examined in all ALDH2 structures (Fig 2A–2E). A particular feature of this catalytic domain is its projection outward from the structure. Furthermore, residues involved to biological processes such as interactions between proteins and to ligands have higher soluble accessible solvent area, whereas scaffolding residues (structure and folding stability) are core internal residues in the protein.

The electrostatic surface potentials were generated through The Adaptive Poisson-Boltzmann Solver (APBS) package [13, 32, 33] for representative proteins of the tomato ALDH2 and ALDH3 families (Figs 2 and 3). In order to differentiate family members of ALDH 2 and 3, we analyzed the positive and negative charges distribution in the surface of the protein models generated. The color in the models depicts the differential properties of the surface, where red color represent negative charges, blue positive and white neutral. The proteins are depicted by two surface views rotated 180° around the vertical (Z) axis. We found that the overall topologic structure is comparable (except for ALDH2B7), several differences can still be observed. A specific positive electrostatic potential distribution dominates the oligomerization domain surface, which is integrate the largest number of positive residues, where also included the polymerization region, and spanning to the cofactor binding region. We can hypothesize that these characteristic patterns of charges distribution might associate differential activities. In addition, these differences also indicate differences in the possible functional mechanism and/or interaction with other proteins and subcellular localization. Furthermore, the most significant differences in the charge distribution were found in the catalytic and the cofactor-binding domains.

Crystallographic structures of different ALDHs are characterized by certain degree of conformational flexibility for the NAD+ cofactor that reveals functional dynamic preference for the oxidized or reduced NADH/NAD cofactor [13, 14, 32, 34]. In this study, using homology modelling to build structures of tomato ALDH, provide novel insights about relationship between structural surfaces and the shape of the ALDH catalytic clefts, enabling us to study the important structural features that dictate cofactor specificity—the NAD+ binding pocket (Fig 3) within the family. The differences in the binding pockets variability is a direct reflect of the functional variability of the different families of ALDH. Overall, the different ALDH proteins are known to have variable conformational features that distinguish non-homologous proteins, i.e. variance ligand molecules, or variation in the shapes of binding pockets for the same ligand [35]. Furthermore, ALDH2 and ALDH3 families have a NAD ring more protected and buried deeper in the binding pocket [13, 14, 32]. This feature was noticeable for the NAD-binding patterns of tomato ALDH2 and ALDH3 [36].

The conservational analysis of the residues included in the substrate and cofactor binding sites, and the structural comparisons of NADP+-dependent ALDHs with known NADP+-dependent isoforms are crucial for predicting the cofactor specificity and the enzymatic mechanism (Fig 3A). We found a conserved Glu residue in different positions and located in the opposite side of the NAD ring, and other conserved Cys residue. Both conserved residues are implicated in the enzymatic mechanism of the ALDH, particularly in the nucleophilic attack and proton abstraction from the Cys during the course of the reaction (Fig 3A–3C). This is also a crucial feature that influences the thiol extraction step during catalysis by the different ALDHs [37].

The tomato ALDH2 family members’ comparison revealed that their substrate-binding sites are similar and are formed by an aromatic cluster mainly composed of phenylalanine, tryptophan residues and several nonpolar residues. These comparisons also revealed that the different residues included in the co-enzyme and catalytic domains (Fig 3A–3C) are well conserved, and for extension the catalytic environment as show the table of amino acids in Fig 3. All member of ALDH2 conserve the distinctive couple of E and C residues in different positions, and key amino acids making the catalytic and coenzyme cleft. However the largest differences in these amino acids were found for ALDH2B7.

ALDH3 superfamily was structure-functionally analyzed (Figs 4 and 5). We found a commonly shared feature when compared to ALDH2 superfamily, which is a specific positive electrostatic potential distribution more extended in the oligomerization domain surface for ALDH3H1 (Fig 4A), and ALDH3Fd1 (Fig 5B), which also extend over the cofactor binding domain. Surprisingly, ALDH3F1 is missing the oligomerization domain and part of the co-enzymatic domain (Fig 5A), which for the best of our knowledge this is the first time that it has been described this structural feature for the superfamily ALDH3 in plants. Sometime, during the evolution process, especially when an organism change the living environment, duplicated genes may undergo divergent fates such as non-functionalization (loss of original functions), neo-functionalization (achievement of novel functions), or sub-functionalization (partition of original functions) [38, 39].

Functional analysis of this ALDH3 superfamily has showed that ALDH3H1 has a conservative residues environment for the co-enzymatic (Fig 4B and 4C) including residues as W113 binding to NAD phosphate, E140 interacting to pentose ring and F340 to nicotinic ring; and catalytic (Fig 4B) cleft environment integrated by T186, G187, E209, L210, E338 and F340, and where key residues involved in the catalytic reaction are C244 and N114 (Fig 4D).

Analysis of the electrostatic potential of ALDH3F1b and ALDH3F1d co-enzyme and catalytic domains showed a similar distribution of surface charges in both proteins (Fig 5A and 5B). A superimposition analysis (Fig 5C) between ALDH3F1b and ALDH3F1d also showed a completely missed oligomerization domain, as well as few a-helices of the coenzyme domain for ALDH3F1b, highlighted in the figure by black arrows. However, and beside these missed structural elements, this ALDH variant is able to accommodate the NAD+ coenzyme, and conserves most of the residues integrating the catalytic domain as E94, E213, the driving catalytic reaction C132, but it is missing the other catalytic residue (asparagine), which is present in tomato ALDH3F1d (N116) (Fig 5E). This feature may indicate that tomato ALDH3F1b is not a functional protein since i) native FALDH is only active as a dimer [40, 41], and ALDH3F1b is missing the oligomerization domain; and ii) this tomato ALDH may not be able to perform the catalytic reaction (the oxidation of long-chain fatty aldehydes), since missing the key reactive asparagine. This residue’s proposed mechanism in the ALDH3H or ALDH3F consists in the activation of C249 by a base (possibly E343), initiating a nucleophilic attack on the carbonyl carbon of the aldehyde. Correct positioning of the polar aldehyde head group is supported by N116 (missing in tomato ALDH3F1b), and an oxyanion liberate a hydride ion, which is transferred to NAD. After that, a proton is transferred from a water molecule, which initiates a nucleophilic attack on the carbonyl carbon of the covalently bound substrate, so an oxyanion breaks the thio-hemiacetal bond and releases the fatty acid product [42].

### Expression analysis of tomato ALDH genes involved in stress response

ALDH genes are in a cross-road stress response situation and represent one of the most important gene superfamily in plants for adaptation to several stresses [43].

Reducing the detrimental effects by decreasing ROS levels through both enzymatic and no-enzymatic pathways seem to represent an important stress-tolerant trait. Crop growth and yield could be treated by stresses, since ROS produced by cells under abiotic and biotic stresses would directly react with proteins, amino acids, and nucleic acids, and cause oxidative damage (peroxidation) of membrane lipids. Thus, levels of aldehydes and ROS molecules in cells must be well balanced, since rapid and high levels of ROS generation would be an important component of the resistance response (oxidative burst) of plants to pathogen attack. On the other hand, intermediate or moderate levels of ROS may serve as direct protective agents by their toxicity or by their ability to confront pathogen invasion [44].

It has been reported that wounding is a main trigger besides osmotic (salt and dehydration) stress for ALDH7B4 (antiquitin) induction in Arabidopsis, where wounding and osmotic stress share signaling pathway [45]. Furthermore, ALDH7B4 may also be involved in response to plant pathogens.

To gain insight into the expression patterns of tomato ALDH2B7 gene in leaf tissue, we used Affymetrix (GDS1670/Clef46b1/aw931836) microarray dataset generated on the same platform (GPL788) by Robert Fluhr [46] that compare WT and Rboh-inhibited mutant plants. Rboh (the respiratory burst oxidase homolog genes) seem to play critical roles in plant development, defense and hormone signaling [47, 48]. This family encodes the key enzymatic subunit of the plant NADPH oxidase, a superoxide-generating enzyme, also identified in different plant species [49]. Plant Rbohs mediate many different responses to stimuli such as development signals [50], and abiotic stresses [51].

We analyzed the response to wounding stress in WT and Rboh- induced mutant plants of *S*. *lycopersicum* ALDH2B7, by mining a publicly available ten tomato microarray datasets. A barr diagram has been depicted of ALDH2B7 expression was presented in S1 Fig. Expression levels of ALDH2B7 were significantly altered under wounding stress in comparison to control plants, and when compared WT to Rboh-inhibited mutant plants (S1 Fig). Among these microarrays, down-regulation seems to be consistent in WT/control plants. However, a significant increase of ALDH2B7 gene expression (up-regulation) occurs when WT plants are wounded. This is in agreement with microarray data from ALDH in *Populus trichocarpa* [25] where 12 genes where up-regulated at 1 week after wounding. Furthermore, transcripts levels of the genes PtALDH2B4, 2B6, 3J1, 3H1, and 3H4 were raised up at 90 hours after root tips wounding. These changes in response to wounding stress pointed out the possible functional divergence of PtALDHs. On the other hand, *S*. *lycopersicum* ALDH2B7 gene expression is reduced when Rboh-inhibited mutant plants are wounded in comparison to controls experiments. This small decrease may be compatible with decrease in the levels of Rboh protein in these *S*. *lycopersicum* plants, since lower quantity of ROS would be generated in these plants, thus lower levels of ROS “scavengers” as ALDH would be required to balance the wounding stress situation.

In summary, and based in the array expression data available of the tomato ALDH families, and their functional and stresses implications, structure-functional characterization of ALDH members of these families provides important knowledge for future improvements of crop stress tolerance. Thus, the regulation of plant stress-related genes expression as ALDH superfamily seems suitable strategy to be used to increase crop stress tolerance. Moreover, this array datasets analysis highlighted the potential roles of ALDH genes in maintaining the balance of ROS and aldehyde species in plant responses to wounding stress, i.e. by pathogen attack. Further functional studies would also be required to examine alternative activities of tomato ALDHs in developmental processes and stress tolerance.

## Conclusions

The ALDHs represent a gene superfamily encoding NAD(P)+-dependent enzymes involved in endogenous and exogenous aldehyde metabolism that catalyze the irreversible oxidation of a broad range of highly reactive aromatic and aliphatic aldehydes to carboxylic acids. The ALDH gene superfamily has been identified and reviewed in different organisms including plants, but no systematic and structure-functional analyses have been made to date in tomato, a model plant for fruit development.

In the present study, comprehensive analyses including tomato genome analysis, ALDH genes identification and naming, comparative phylogeny, structure-functional analysis of ALDH2 and ALDH3 families, and ALDH genes expression in developmental tissues and under different stresses were performed. A total of 29 tomato ALDH genes have been identified in the *S*. *lycopersicum* genome. They were grouped into 11 families providing a unified nomenclature for the deduced ALDH polypeptides based in the criteria of the ALDH Gene Nomenclature Committee (AGNC). An ALDH19 gene is identified as unique among plants ALDH. Phylogenetic analysis indicates that ALDHs of tomato were split into two small and one big clade, but these are divided in a total number of 13 groups where different ALDH families were well grouped with Arabidopsis ALDH families. Analyses demonstrate that organization of ALDH families, sub-cellular distribution based on other species ALDH gene families, structure-functional features and expression profiles of ALDH genes are fairly conserved in tomato. However, some duplication variants for tomato ALDH2, ALDH3, ALDH5, ALDH7 and ALDH11 may be responsible to cope with some stresses, although some of these variants were generated with non-functionality or without the expected function as ALDH3F1b. Available information about tomato ALDH2B7 gene expression indicate its role in buffering and keeping a good balance in ROS and aldehyde species generation in response to wounding stress.

## Materials and Methods

### Database searches for ALDH genes identification in *S*. *lycopersicum*

To identify the *S*. *lycopersicum* ALDH protein superfamily, Arabidopsis-, rice-, maize-, grape-, soybean, Sorghum bicolor,- *Selaginella moellendorffii*-, poplar-,moss-, algae-, and *O*. *tauri*-ALDH sequences identified previously were retrieved from NCBI (http://www.ncbi.nlm.nih.gov/), and used to investigate ALDH and ALDH-like DNA sequences of *S*. *lycopersicum* with BLASTN, TBLASTN, and BLASTX in BLAST. Annotation details of the *S*. *lycopersicum* genome is available from International Tomato Genome Sequencing Project (https://solgenomics.net/organism/Solanum_lycopersicum/genome), which were scanned with BLAST (http://blast.ncbi.nlm.nih.gov/Blast.cgi) to check sequences from last release annotations. All sequences with an E-value <1e-6 were manually analyzed. The confirmation of the protein motifs of *S*. *lycopersicum* ALDHs were done with the Pfam00171 (ALDH family), PS00687 (ALDH glutamic acid active site), PS00070 (ALDH cysteine active site); and superfamily domains were confirmed with KOG2450, KOG2451, KOG2453, and KOG2456. Potential molecular functions were assigned based on similarities in alignments. *S*. *lycopersicum* ALDH were further annotated on the basis of the annotation criteria of AGNC [52], grouping sequences in families being more than 40% identical to other previously identified ALDH sequences. Sequences with greater than 60% identical were grouped as a protein subfamily. Amino acid sequences less than 40% identical were depicted a new ALDH protein family as previously described [13, 32].

Orthologous functions where identified using OrthoDB database [53].

### Phylogenetic analyses of ALDH gene sequences from tomato and comparison with the model plant Arabidopsis

In order to develop a comparative phylogenetic analysis of *S*. *lycopersicum* ALDH proteins, multiple protein alignments of ALDH protein sequences from *S*. *lycopersicum* and A. thaliana were made using ClustalW multiple sequence alignment tools (http://www.ebi.ac.uk/Tools/clustalw/) using a Blosum32 protein weight matrix, as well as multiple alignment gap opening/extension penalties of 10/0.5 and pairwise gap opening/extension penalties of 10/0.1.

BioEdit V 7.1.3.0 was used for alignments analysis, where portions of sequences that did not aligned with high confidence were removed. Neighbor-joining (NJ) method was used for phylogenetic studies. Branches of the tree were tested with 1000 bootstrap replicates. Trees were visualized by using Treedyn (www.treedyn.org)

### Expression analysis of tomato ALDH2B7 gene based in microarray data analyses

We perform an exhaustive searching in different database (Web of Science, PubMed, NCBI, and Uniprot) about the microarray data available of the expression of ALDH superfamily in tomato in response to different biotic and abiotic stresses, and affecting different plant organs and in different stages of development. So far, there are not large studies measuring the importance of this superfamily.

The microarray data concerning the expression of ALDH2B7 in *S*. *lycopersicum* was available at NCBI Gene Expression Omnibus (GEO) database [54], and retrieved with the series accession numbers GSM13872 to GSM13881. These were found searching in the NCBI GEO database using GEO BLAST tool (http://blast.ncbi.nlm.nih.gov/Blast.cgiPROGRAM=blastn&BLAST_SPEC=GeoBlast&PAGE_TYPE=BlastSearch). No other array was found NCBI GEO or other database about *S*. *lycopersicum* ALDH expression under different stresses conditions. The arrays correspond to the serie GSE917 (Systemic Leaf Wound Response in Tomato), GDS1670/cLEF46B11/AW931836.

GSM13877 to GSM13881 include microarray data from leaf tissue samples representing three biological replicates in WT plants, whereas series GSM13872 to GSM13876 contains microarray data from leaf tissue samples representing three biological replicates in Rboh-inhibited mutant plants.

### ALDH proteins modelling and structural features study

The ALDH protein sequences (ALDH2B1, ALDH2B4, ALDH2B7, ALDH2C4, and ALDH3F1) were used searching for homology in the Protein Data Bank (PDB). The homologous templates suitable for these sequences were selected by BLAST server (http://ncbi.nlm.nih.gov/). BioInfoBank Metaserver (http://meta.bioinfo.pl/), a fold recognition homology was also used for templates selection. Furthermore, the results obtained by previous methods were also compared with these obtained by Swiss-model server for template identification (swissmodel.expasy.org). The best templates, 1ad3 [55], 4qgk [41], 4pxl [56], were retrieved from PDB database and used for homology modelling.

ALDH protein models were built using the top PDB closed template structures by SWISS-MODEL via the ExPASy web server (swissmodel.expasy.org).

An initial structural model generated was assessed for recognition of errors in 3D structure by using ProSA (prosa.services.came.sbg.ac.at/prosa.php), and also for a first overall quality estimation of the model with QMEAN (swissmodel.expasy.org/qmean/cgi/index.cgi). Final structures of ALDH proteins were subjected to energy minimization using GROMOS96 and implemented in DeepView/Swiss-PDBViewer v3.7 (spdbv.vital-it.ch) to improve the van der Waals contacts and correct the stereochemistry of the model.

The quality of the model was assessed by QMEAN, testing proteins stereology with PROCHECK (www.ebi.ac.uk/thornton-srv/software/PROCHECK), and ProSA (prosa.services.came.sbg.ac.at/prosa.php) programs, as well as the protein energy with ANOLEA (protein.bio.puc.cl/cardex/servers/anolea).

The Ramachandran plot statistics for the models were also calculated to show the number of protein residues in the favored regions.

The electrostatic Poisson-Boltzmann (PB) potentials for all the structures were analyzed using APBS (DeLano Scientific LLC) molecular modelling software implemented in PyMOL 0.99 (www.pymol.org). Potential values are given in units of kT per unit charge (k Boltzmann’s constant; T temperature).

## Supporting Information

S1 Fig Expression level of ALDH2B7 in *S*. *lycopersicum* leaf tissue under abiotic stress. Tomato WT and the respiratory burst oxidase homolog genes (Rboh)—inhibited mutant plants were used to check the aldehyde dehydrogenase SlALDH2B7 gene expression variation under wounding stress by mean of microarray available data series GSE917 at NCBI Gene Expression Omnibus (GEO) database.
(TIF)

S1 Table ALDH family members identified in plants. Tomato ALDH members of the different families have been compared to ALDH families previously identified in seventeen species of plants (monocots and dicots), algae, and mosses.
(DOCX)
