# Supplementary figures and images for: Genome-Wide Identification and Functional Classification of Tomato (Solanum lycopersicum) Aldehyde Dehydrogenase (ALDH) Gene Superfamily
Source: PLoS One. 2016 Oct 18;11(10):e0164798. doi: 10.1371/journal.pone.0164798 (PMC5068750; doi:10.1371/journal.pone.0164798)

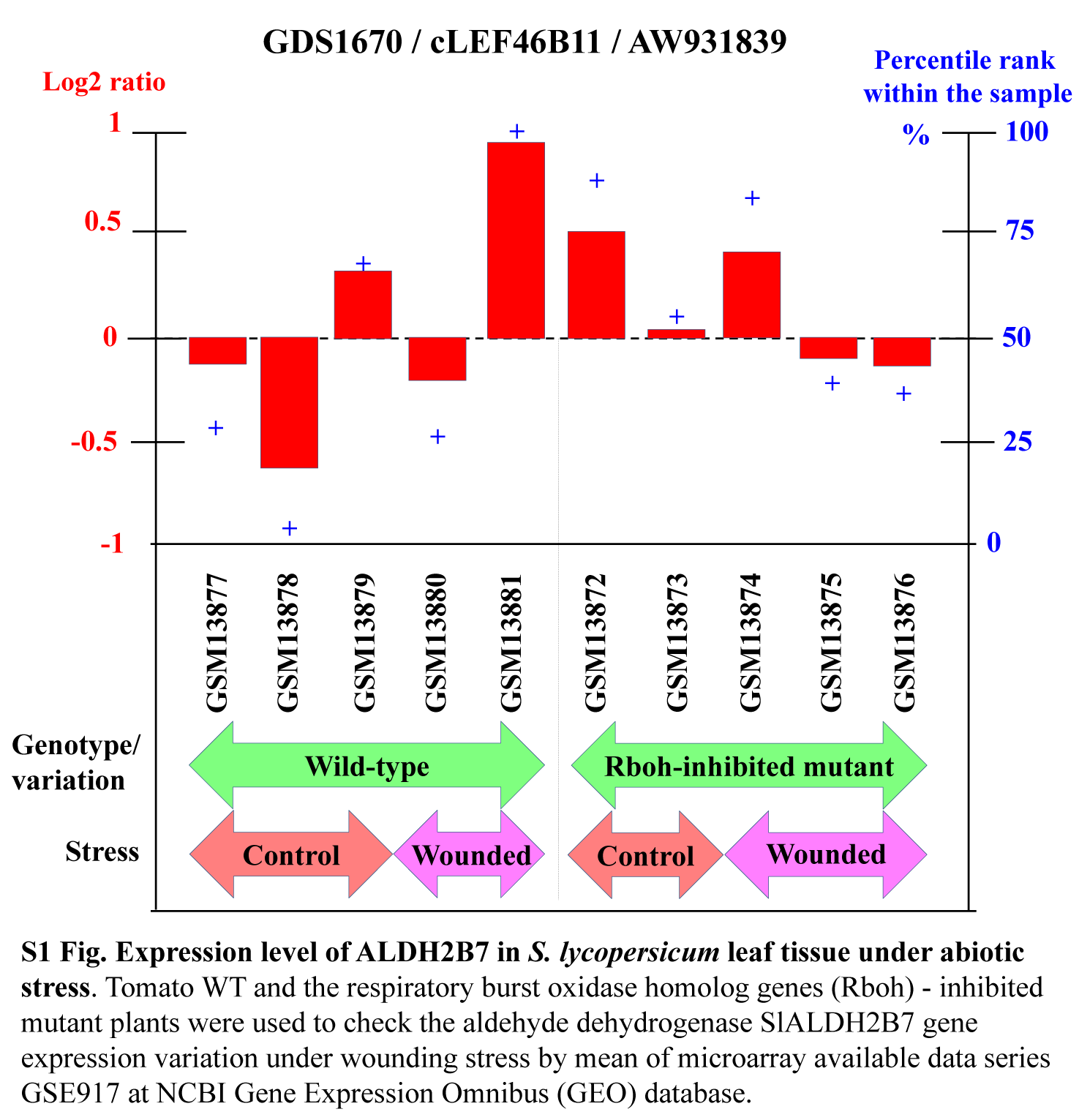

Supplement: S1 Fig — Tomato WT and the respiratory burst oxidase homolog genes (Rboh)—inhibited mutant plants were used to check the aldehyde dehydrogenase SlALDH2B7 gene expression variation under wounding stress by mean of microarray available data series GSE917 at NCBI Gene Expression Omnibus (GEO) database. (TIF) [file pone.0164798.s001.tif]
