# Supplementary material for: Genome-Wide Identification and Functional Classification of Tomato (Solanum lycopersicum) Aldehyde Dehydrogenase (ALDH) Gene Superfamily
Source: PLoS One. 2016 Oct 18;11(10):e0164798. doi: 10.1371/journal.pone.0164798 (PMC5068750; doi:10.1371/journal.pone.0164798)
Supplement: S1 Table — Tomato ALDH members of the different families have been compared to ALDH families previously identified in seventeen species of plants (monocots and dicots), algae, and mosses. (DOCX) [file pone.0164798.s002.docx]

**S1 Table. ALDH family members identified in plants.** Tomato ALDH members of the different families have been compared to ALDH families previously identified in seventeen species of plants (monocots and dicots), algae, and mosses.

| **Specie** | **Common name** | **2** | **3** | **4** | **5** | **6** | **7** | **8** | **9** | **10** | **11** | **12** | **13** | **14** | **15** | **16** | **17** | **18** | **19** | **20** | **21** | **22** | **23** | **24** | **ALDH number** | **Reference** |
| --- | --- | --- | --- | --- | --- | --- | --- | --- | --- | --- | --- | --- | --- | --- | --- | --- | --- | --- | --- | --- | --- | --- | --- | --- | --- | --- |
| *Solanum lycopersicum* | Tomato | 8 | 5 | - | 2 | 1 | 2 | - | - | 2 | 4 | 1 | - | - | - | - | - | 2 | 1 | - | - | 1 | - | - | 29 | Current study |
| *Arabidopsis thaliana* | Thale cress | 3 | 3 | - | 1 | 1 | 1 | - | - | 2 | 1 | 1 | - | - | - | - | - | 2 | - | - | - | 1 | - | - | 16 | [11] |
| *Oryza sativa* | Asian rice | 5 | 5 | - | 1 | 1 | 1 | - | - | 2 | 1 | 1 | - | - | - | - | - | 2 | - | - | - | 1 | - | - | 20 | [8] |
| *Zea mays* | Maize | 6 | 5 | - | 2 | 1 | 1 | - | - | 3 | 1 | 1 | - | - | - | - | - | 2 | - | - | - | 1 | - | - | 23 | [14] |
| *Glycine max* | Soybean | 5 | 1 | - | - | - | 4 | - | - | 6 | 2 | - | - | - | - | - | - | - | - | - | - | - | - | - | 18 | [15] |
| *Setaria italica* | Foxtail Millet | 6 | 4 |  | 1 | 1 | 1 |  |  | 2 | 1 | 1 |  |  |  |  |  | 2 |  |  |  | 1 |  |  | 20 | [49] |
| *Chlamydomonas reinhardtii* | Unicellular green algae | 1 | - | - | 1 | 1 | - | - | - | 1 | 1 | 1 | - | - | - | - | - | 1 | - | - | - | 1 | - | 1 | 9 | [12] |
| *Physcomitrella patens* | Moss | 2 | 5 | - | 2 | 1 | 1 | - | - | 1 | 5 | 1 | - | - | - | - | - | 1 | - | - | 1 |  | 1 | - | 21 | [12] |
| *Populus trichocarpa* | Black cottonwood | 4 | 6 | - | 1 | 4 | 2 | - | - | 2 | 3 | 1 | - | - | - | - | - | 2 | - | - | - | 1 | - | - | 26 | [25] |
| *Selaginella moellendorffii* | Gemmiferous Spikemoss | 6 | 2 | - | 1 | 1 | 1 | - | - | 1 | 6 | 1 | - | - | - | - | - | 1 | - | - | 1 | 1 | 2 | - | 24 | [10] |
| *Sorghum bicolor* | Sorghum | 5 | 4 | - | 1 | 1 | 1 | - | - | 2 | 1 | 1 | - | - | - | - | - | 2 | - | - | - | 1 | - | - | 19 | [59] |
| *Gossypium raimondii* | New World Cotton | 8 | 6 | - | 1 | 3 | 1 | - | - | 2 | 3 | 1 | - | - | - | - | - | 4 | - | - | - | 1 | - | - | 30 | [60] |
| *Malus domestica* | Apple | 13 | 7 | - | 2 | 2 | 2 | - | - | 2 | 3 | 2 | - | - | - | - | - | 4 | - | - | - | 2 | - | - | 39 | [17] |
| *Eutrema parvulum* | Algae | 3 | 3 | - | 1 | 1 | 1 | - | - | 2 | 1 | 1 | - | - | - | - | - | 2 | - | - | - | 1 | - | - | 16 | [7] |
| *Eutrema salsugineum* | Algae | 3 | 4 | - | 1 | 1 | 1 | - | - | 2 | 1 | 1 | - | - | - | - | - | 2 | - | - | - | 1 | - | - | 16 | [7] |
| *Vitis vinifera* | Common wine grape | 5 | 4 | - | 3 | 3 | 2 | - | - | 2 | 2 | 1 | - | - | - | - | - | 2 | - | - | - | 1 | - | - | 25 | [16] |
| *Volvox carteri* | Colonial green algae | 1 | - | - | - | 1 | - | - | - | 1 | 1 | 1 | - | - | - | - | - | 1 | - | - | - | 1 | - | - | 7 | [61] |
| *Ostreococcus tauri* | Unicellular green algae | - | 1 | - | 1 | - | - | - | - | 1 | 1 | 1 | - | - | - | - | - | - | - | - | - | 1 | - | - | 6 | [12] |

**S1 Table References**

7. Hou Q, Bartels D (2015) Comparative study of the aldehyde dehydrogenase (ALDH) gene superfamily in the glycophyte Arabidopsis thaliana and Eutrema halophytes. Ann Bot 115: 465-479

8. Gao C, Han B (2009) Evolutionary and expression study of the aldehyde dehydrogenase (ALDH) gene superfamily in rice (Oryza sativa). Gene 431: 86-94

10. Brocker C, Vasiliou M, Carpenter S, Carpenter C, Zhang Y, Wang X et al. (2013) Aldehyde dehydrogenase (ALDH) superfamily in plants: gene nomenclature and comparative genomics. Planta 237: 189-210

11. Kirch H-H, Bartels D, Wei Y, Schnable PS, Wood AJ (2004) The ALDH gene superfamily of Arabidopsis. Trends Plant Sci 9: 371-377

12. Wood AJ, Duff RJ (2009) The aldehyde dehydrogenase (ALDH) gene superfamily of the moss Physcomitrella patens and the algae Chlamydomonas reinhardtii and Ostreococcus tauri. The Bryologist 112: 1-11

14. Jimenez-Lopez JC, Gachomo EW, Seufferheld MJ, Kotchoni SO (2010) The maize ALDH protein superfamily: linking structural features to functional specificities. BMC Struct Biol 10: 43

15. Kotchoni SO, Jimenez-Lopez JC, Kayodé APP, Gachomo EW, Baba-Moussa L (2012) The soybean aldehyde dehydrogenase (ALDH) protein superfamily. Gene 495: 128-133

16. Zhang Y, Mao L, Wang H, Brocker C, Yin X, Vasiliou V et al. (2012) Genome-wide identification and analysis of grape aldehyde dehydrogenase (ALDH) gene superfamily. PLoS One 7: e32153

17. Li X, Guo R, Li J, Singer SD, Zhang Y, Yin X et al. (2013) Genome-wide identification and analysis of the aldehyde dehydrogenase (ALDH) gene superfamily in apple (Malus × domestica Borkh.). Plant Physiol Biochem 71: 268-282

25. Tian F-X, Zang J-L, Wang T, Xie Y-L, Zhang J, Hu J-J (2015) Aldehyde Dehydrogenase Gene Superfamily in Populus: Organization and Expression Divergence between Paralogous Gene Pairs. PLoS One 10: e0124669

49. Cheng C, Xu X, Gao M, Li J, Guo C, Song J, et al. (2014) Genome-Wide Analysis of Respiratory Burst Oxidase Homologs in Grape (Vitis vinifera L.). Int J Mol Sci 14: 24169-24186

59. Paterson AH, Bowers JE, Bruggmann R, Dubchak I, Grimwood J, Gundlach H et al. (2009) The Sorghum bicolor genome and the diversification of grasses. Nature 457: 551–556

60. He D, Lei Z, Xing H, Tang B (2014) Genome-wide identification and analysis of the aldehyde dehydrogenase (ALDH) gene superfamily of *Gossypium raimondii*. Gene 549: 123–133

61. Prochnik SE, Umen J, Nedelcu AM, Hallmann A, Miller SM, Nishii I et al. (2010) Genomic analysis of organismal complexity in the multicellular green alga Volvox carteri. Science 329: 223–226
